# Advanced Eco-Friendly Wood-Based Composites

**DOI:** 10.3390/ma15238651

**Published:** 2022-12-05

**Authors:** Roman Reh, Lubos Kristak, Petar Antov

**Affiliations:** 1 Faculty of Wood Sciences and Technology, Technical University in Zvolen, 960 01 Zvolen, Slovakia; 2 Faculty of Forest Industry, University of Forestry, 1797 Sofia, Bulgaria

In collaboration with the MDPI publishing house, we are pleased to introduce the reader to our new project, the Special Issue entitled “Advanced Eco-friendly Wood-Based Composites”. This Special Issue provides an opportunity to investigate the advanced eco-friendly wood-based composites from a broader perspective. The coronavirus pandemic and shutdown measures employed to contain it, as well as the ongoing war, have influenced and decelerated the world economy and adversely impacted the research activities on most levels in all countries. Surprisingly, researchers in the field of wood-based composites have continued to make progress, which is also described in this Special Issue.

The wood of forest trees is a renewable, sustainable and easily workable material and has been widely used in construction, paper making, and furniture and as a feedstock for biofuels. Wood composites are engineered wood-based materials that are fabricated from a wide variety of wood and other non-wood lignocellulosic materials, bonded with synthetic or natural bio-based adhesive systems, and designed for specific value-added applications and performance requirements [1,2,3,4,5,6]. Traditional wood-based composites are fabricated using synthetic formaldehyde-based adhesives that are commonly formed from fossil-derived constituents, such as urea, phenol, and melamine [7,8,9]. Along with their undisputable advantages, these adhesives are characterized by certain problems related to the emission of hazardous volatile organic compounds (VOCs), including free formaldehyde emissions from the finished wood composites, which is carcinogenic to humans and harmful to the environment [10,11,12]. The growing environmental concerns connected with the adoption of circular economy principles and the new, stricter legislative requirements for the emission of harmful VOCs, such as free formaldehyde, from wood composites pose new challenges for researchers and industrial practice. These challenges are related to the development of sustainable, eco-friendly wood composites [13,14,15], the optimization of the available lignocellulosic raw materials [16,17,18], and the use of alternative resources [19,20,21,22,23]. The harmful release of formaldehyde from wood composites can be reduced by applying formaldehyde scavengers to conventional adhesive systems [24,25,26,27], by the surface treatment of the finished wood composites, or by the application of novel bio-based wood adhesives as environmentally friendly alternatives to traditional synthetic resins [28,29,30]. Another alternative to the use of synthetic formaldehyde-based adhesives is the manufacturing of binderless wood composites, since wood is a natural polymer material that is rich in lignocellulosic compounds such as cellulose, hemicellulose, and lignin.

This Special Issue represents a collection of 11 high-quality original research and review papers that provide examples of the latest advancements in the development and applications of eco-friendly wood-based composites.

In their paper, Bekhta et al. investigated the potential of incorporating lignin-based additives, i.e., magnesium and sodium lignosulfonates, in urea-formaldehyde resin in order to manufacture low-toxic, eco-friendly particleboards with acceptable physical and mechanical properties and achieve reduced formaldehyde emissions [31]. The adhesive system employed by the authors also included polymeric 4,4′-diphenylmethane diisocyanate (pMDI) as a crosslinker. The authors determined that the lignosulfonate addition levels varied from 10 to 30%, resulting in particleboards with physical and mechanical properties comparable to those of panels bonded with UF resin alone. In addition, the panels bonded with lignosulfonates and pMDI exhibited a close-to-zero formaldehyde content, reaching the super E0 emission grade of ≤1.5 mg/100 g.

In another paper, Mirski et al. studied the effect of the structure of lattice beams on their strength properties [32]. Based on the results obtained from the study, it was concluded that the solutions proposed by the authors represent alternatives to wooden trusses, which are joined with flanges using punched metal plate fasteners. However, it should be noted that, at the current stage of this research, these solutions exhibited approximately 30% lower static bending strength values than trusses fabricated with metal plates.

The feasibility of employing novel lightweight panels fabricated from waste corrugated cardboard and beech veneer, as structural materials with applications in interior and furniture construction, was studied by Jivkov et al. [33]. In laboratory conditions, the authors developed two types of multi-layered panels and evaluated the bending moments and stiffness coefficients of seven different types of end corner joints (demountable joints and those fixed with an adhesive) formed from the developed composites. The authors concluded that these materials can be successfully used in the construction of furniture and other interior elements.

Following the circular economy principles, i.e., the reuse, recycling, or upcycling of materials for the purpose of the increased utilization of waste and by-products in value-added applications, Mirski et al. investigated the possibilities of using waste wood particles obtained from the primary wood processing as a filler for polyurethane foams (PUR) with an open-cell structure [34]. It was found that the addition of 10% waste wood particles resulted in 30% increased compressive strength values of the PUR foam and 10% decreased thermal conductivity, respectively. The authors concluded that the developed composite foams can be efficiently used in thermal insulation applications in the construction of prefabricated buildings.

In another interesting study, an attempt was made to predict the mechanical properties, i.e., the modulus of elasticity (MOE) and modulus of rupture (MOR), of artificially weathered fir, alder, oak, and poplar wood by investigating the variations in the color parameters of the wood samples and developing a machine learning model [35]. It was found that the deflection to failure of the wood samples increased with the weathering, which was attributed to the increased viscoelasticity of the weathered wood samples. Significantly, the experimental work was performed only on small-sized, clear wood samples without defects. Thus, the effectiveness of the developed model should be further analyzed using large-sized wood specimens.

Handika et al. reported the isolation of lignin from black liquor, used as a pre-polymer for the preparation of bio-based polyurethane resin, which was exploited for the impregnation of ramie fiber (*Boehmeria nivea* (L.) Gaudich) with the aim of improving its thermal and mechanical properties [36]. One-step fractionation of the isolated lignin was performed using methanol and acetone as solvents. Based on the experimental results, the authors concluded that the increased mechanical properties, i.e., the tensile strength and MOE, as well as the enhanced thermal stability of the impregnated ramie fiber, could expand its future potential for wider industrial application as a sustainable and functional material.

Wronka et al. studied the potential of using raspberry (*Rubus idaeus* L.) and black chokeberry (*Aronia melanocarpa* (Michx.) Elliott) lignocellulosic particles for manufacturing particleboards intended for furniture applications [37]. The authors also characterized the wooden particles, obtained from the re-milling particleboards, in order to evaluate their recycling possibilities. The authors reported the successful fabrication of particleboards from both lignocellulosic by-products. Significantly, the addition of raspberry particles should not exceed 50% in order to obtain boards with mechanical properties that fulfil the European standard requirements. In addition, it was found that the upcycling of the particles obtained from the re-milled panels is rather limited due to the significantly different fractions and shape of particles.

The study carried out by Dukarska et al. aimed to investigate and characterize the physical properties of wood particles intended for the manufacturing of particleboards according to their moisture content [38]. It was found that the increased moisture content of the wood particles resulted in an increase in their dimensions, regardless of their degree of fineness, as well as an increased slippery angle of repose. In addition, the greater moisture content of the particles resulted in an increased tapped bulk density for both types of particles evaluated, e.g., the microparticles of the outer layers of the particleboards and the particles of the core layers of the panels. The results obtained could be of great benefit in the industrial practices of the wood-based panel industry with respect to the optimization of the technological parameters and related production costs.

One of the greatest challenges for the wood composite industry is the increased demand for wood and other lignocellulosic raw materials [39,40,41]. This has led to significantly increased interest in the industrial and research sectors in efforts to identify alternative raw materials as natural feedstocks for the production of wood composites. In their study, Pędzik et al. evaluated the potential of using walnut (*Juglans regia* L.) wood residues as an alternative raw material for the production of particleboards [42]. The authors reported that the mechanical properties of the panels, which were produced in the laboratory with 50% walnut wood particles, fulfilled the European standard requirements for particleboards intended for load-bearing applications.

Exposure to wood dust is one of the greatest occupational hazards to the health and safety of workers in wood-processing and furniture enterprises [43,44,45,46]. The results of the study carried out by Dembiński et al. will be of great benefit for the industrial practice of furniture factories in terms of methods for predicting the separation efficiency in the long-term use of filter bags employed in the wood-based panel industry [47].

Last but not least, a comprehensive review of the possibilities of using hemp as an abundant and renewable natural raw material for the polymer industry was conducted by Tutek and Masek [48]. The authors presented and critically discussed the chemical composition and physical and mechanical properties of hemp fibers, oil, wax, and extracts and provided relevant examples of the use of hemp derivatives in polymer composites.

The ongoing transition of the wood-based panel industry toward a circular, low-carbon bio-economy is a strong prerequisite for the continuous development of sustainable and eco-friendly wood composites. The examples presented herein represent only a selection and short overview of the future research trajectories related to the development, properties, and applications of innovative, high-performance, eco-friendly wood composites with a lower environmental impact.

## References

[1] Irle M.A., Barbu M.C., Réh R., Bergland L., Rowell R.M. (2012). Wood Composites. Handbook of Wood Chemistry and Wood Composites.

[2] Pizzi A., Papadopoulos A.N., Policardi F. (2020). Wood composites and their polymer binders. Polymers.

[3] Krišťák Ľ., Réh R. (2021). Application of Wood Composites. Appl. Sci..

[4] Papadopoulos A.N. (2021). Advances in Wood Composites III. Polymers.

[5] Mirski R., Derkowski A., Kawalerczyk J., Dziurka D., Walkiewicz J. (2022). The Possibility of Using Pine Bark Particles in the Chipboard Manufacturing Process. Materials.

[6] Lee S.H., Lum W.C., Boon J.G., Kristak L., Antov P., Rogoziński T., Pędzik M., Taghiyari H.R., Lubis M.A.R., Fatriasari W. (2022). Particleboard from Agricultural Biomass and Recycled Wood Waste: A Review. J. Mater. Res. Technol..

[7] Mantanis G.I., Athanassiadou E.T., Barbu M.C., Wijnendaele K. (2018). Adhesive systems used in the European particleboard, MDF and OSB industries. Wood Mater. Sci. Eng..

[8] Dorieh A., Ayrilmis N., Pour M.F., Movahed S.G., Kiamahalleh M.V., Shahavi M.H., Hatefnia H., Mehdinia M. (2022). Phenol formaldehyde resin modified by cellulose and lignin nanomaterials: Review and recent progress. Int. J. Biol. Macromol..

[9] Barbu M.C., Irle M., Réh R., Aguilera A., Davim P. (2014). Wood Based Composites. Research Developments in Wood Engineering and Technology.

[10] Kumar R.N., Pizzi A. (2019). Environmental Aspects of Adhesives–Emission of Formaldehyde. Adhesives for Wood and Lignocellulosic Materials.

[11] Walkiewicz J., Kawalerczyk J., Mirski R., Dziurka D., Wieruszewski M. (2022). The Application of Various Bark Species as a Fillers for UF Resin in Plywood Manufacturing. Materials.

[12] Bekhta P., Sedliačik J., Noshchenko G., Kačík F., Bekhta N. (2021). Characteristics of Beech Bark and its Effect on Properties of UF Adhesive and on Bonding Strength and Formaldehyde Emission of Plywood Panels. Eur. J. Wood Prod..

[13] Ninikas K., Mitani A., Koutsianitis D., Ntalos G., Taghiyari H.R., Papadopoulos A.N. (2021). Thermal and Mechanical Properties ofGreen Insulation Composites Made from Cannabis and Bark Residues. J. Compos. Sci..

[14] Antov P., Savov V., Trichkov N., Krišťák Ľ., Réh R., Papadopoulos A.N., Taghiyari H.R., Pizzi A., Kunecová D., Pachikova M. (2021). Properties of High-Density Fiberboard Bonded with Urea–Formaldehyde Resin and Ammonium Lignosulfonate as a Bio-Based Additive. Polymers.

[15] Savov V., Valchev I., Antov P., Yordanov I., Popski Z. (2022). Effect of the Adhesive System on the Properties of Fiberboard Panels Bonded with Hydrolysis Lignin and Phenol-Formaldehyde Resin. Polymers.

[16] Kminiak R., Orlowski K.A., Dzurenda L., Chuchala D., Banski A. (2020). Effect of Thermal Treatment of Birch Wood by SaturatedWater Vapor on Granulometric Composition of Chips from Sawing and Milling Processes from the Point of View of Its Processing to Composites. Appl. Sci..

[17] Reinprecht L., Iždinský J. (2022). Composites from Recycled and Modified Woods—Technology, Properties, Application. Forests.

[18] Pędzik M., Kwidziński Z., Rogoziński T. (2022). Particles from Residue Wood-Based Materials from Door Production as an Alternative Raw Material for Production of Particleboard. Drv. Ind..

[19] Barbu M.C., Sepperer T., Tudor E.M., Petutschnigg A. (2020). Walnut and Hazelnut Shells: Untapped Industrial Resources and Their Suitability in Lignocellulosic Composites. Appl. Sci..

[20] Kain G., Morandini M., Stamminger A., Granig T., Tudor E.M., Schnabel T., Petutschnigg A. (2021). Production and Physical–Mechanical Characterization of Peat Moss (Sphagnum) Insulation Panels. Materials.

[21] Barbu M.C., Montecuccoli Z., Förg J., Barbeck U., Klímek P., Petutschnigg A., Tudor E.M. (2021). Potential of Brewer’s Spent Grain as a Potential Replacement of Wood in pMDI, UF or MUF Bonded Particleboard. Polymers.

[22] Rammou E., Mitani A., Ntalos G., Koutsianitis D., Taghiyari H.R., Papadopoulos A.N. (2021). The Potential Use of Seaweed (*Posidonia oceanica*) as an Alternative Lignocellulosic Raw Material for Wood Composites Manufacture. Coatings.

[23] Pędzik M., Janiszewska D., Rogoziński T. (2021). Alternative Lignocellulosic Raw Materials in Particleboard Production: A Review. Ind. Crops Prod..

[24] Kristak L., Antov P., Bekhta P., Lubis M.A.R., Iswanto A.H., Reh R., Sedliacik J., Savov V., Taghiayri H., Papadopoulos A.N. (2022). Recent Progress in Ultra-Low Formaldehyde Emitting Adhesive Systems and Formaldehyde Scavengers in Wood-Based Panels: A Review. Wood Mater. Sci. Eng..

[25] Mirski R., Kawalerczyk J., Dziurka D., Siuda J., Wieruszewski M. (2020). The Application of Oak Bark Powder as a Filler for Melamine-Urea-Formaldehyde Adhesive in Plywood Manufacturing. Forests.

[26] Medved S., Gajsek U., Tudor E.M., Barbu M.C., Antonovic A. (2019). Efficiency of bark for reduction of formaldehyde emission fromparticleboards. Wood Res..

[27] Kawalerczyk J., Walkiewicz J., Woźniak M., Dziurka D., Mirski R. (2022). The effect of urea-formaldehyde adhesive modification with propylamine on the properties of manufactured plywood. J. Adhes..

[28] Arias A., González-Rodríguez S., Vetroni Barros M., Salvador R., de Francisco A.C., Piekarski C.M., Moreira M.T. (2021). Recent developments in bio-based adhesives from renewable natural resources. J. Clean. Prod..

[29] Maulana M.I., Lubis M.A.R., Febrianto F., Hua L.S., Iswanto A.H., Antov P., Kristak L., Mardawati E., Sari R.K., Zaini L.H. (2022). Environmentally Friendly Starch-Based Adhesives for Bonding High-Performance Wood Composites: A Review. Forests.

[30] Saud A.S., Maniam G.P., Rahim M.H.A., Jawaid M., Khan T.A., Nasir M., Asim M. (2021). Introduction of Eco-Friendly Adhesives: Source, Types, Chemistry and Characterization. Eco-Friendly Adhesives for Wood and Natural Fiber Composites.

[31] Bekhta P., Noshchenko G., Réh R., Kristak L., Sedliačik J., Antov P., Mirski R., Savov V. (2021). Properties of Eco-Friendly Particleboards Bonded with Lignosulfonate-Urea-Formaldehyde Adhesives and pMDI as a Crosslinker. Materials.

[32] Mirski R., Matwiej Ł., Dziurka D., Chuda-Kowalska M., Marecki M., Pałubicki B., Rogoziński T. (2021). Influence of the Structure of Lattice Beams on Their Strength Properties. Materials.

[33] Jivkov V., Simeonova R., Antov P., Marinova A., Petrova B., Kristak L. (2021). Structural Application of Lightweight Panels Made of Waste Cardboard and Beech Veneer. Materials.

[34] Mirski R., Dukarska D., Walkiewicz J., Derkowski A. (2021). Waste Wood Particles from Primary Wood Processing as a Filler of Insulation PUR Foams. Materials.

[35] Nasir V., Fathi H., Fallah A., Kazemirad S., Sassani F., Antov P. (2021). Prediction of Mechanical Properties of Artificially Weathered Wood by Color Change and Machine Learning. Materials.

[36] Handika S.O., Lubis M.A.R., Sari R.K., Laksana R.P.B., Antov P., Savov V., Gajtanska M., Iswanto A.H. (2021). Enhancing Thermal and Mechanical Properties of Ramie Fiber via Impregnation by Lignin-Based Polyurethane Resin. Materials.

[37] Wronka A., Robles E., Kowaluk G. (2021). Upcycling and Recycling Potential of Selected Lignocellulosic Waste Biomass. Materials.

[38] Dukarska D., Rogoziński T., Antov P., Kristak L., Kmieciak J. (2022). Characterisation of Wood Particles Used in the Particleboard Production as a Function of Their Moisture Content. Materials.

[39] Wronka A., Kowaluk G. (2022). Upcycling Different Particle Sizes and Contents of Pine Branches into Particleboard. Polymers.

[40] Wronka A., Beer P., Kowaluk G. (2022). Selected Properties of Single and Multi-Layered Particleboards with the Structure Modified by Fibers Implication. Materials.

[41] Wronka A., Kowaluk G. (2022). The Influence of Multiple Mechanical Recycling of Particleboards on Their Selected Mechanical and Physical Properties. Materials.

[42] Pędzik M., Auriga R., Kristak L., Antov P., Rogoziński T. (2022). Physical and Mechanical Properties of Particleboard Produced with Addition of Walnut (*Juglans regia* L.) Wood Residues. Materials.

[43] Očkajová A., Stebila J., Rybakowski M., Rogoziński T., Krišťák Ľ, Ľuptáková J. (2014). The Granularity of Dust Particles when Sanding Wood and Wood-Based Materials. Adv. Mater. Res..

[44] Igaz R., Kminiak R., Krišťák Ľ., Němec M., Gergeľ T. (2019). Methodology of Temperature Monitoring in the Process of CNC Machining of Solid Wood. Sustainability.

[45] Makovicka Osvaldova L., Petho M. (2015). Occupational Safety and Health During Rescue activities. Procedia Manuf..

[46] Očkajová A., Kučerka M., Kminiak R., Rogoziński T. (2020). Granulometric composition of chips and dust produced from the process of working thermally modified wood. Acta Facultatis Xylologiae Zvolen.

[47] Dembiński C., Potok Z., Kučerka M., Kminiak R., Očkajová A., Rogoziński T. (2022). The Dust Separation Efficiency of Filter Bags Used in the Wood-Based Panels Furniture Factory. Materials.

[48] Tutek K., Masek A. (2022). Hemp and Its Derivatives as a Universal Industrial Raw Material (with Particular Emphasis on the Polymer Industry)—A Review. Materials.

